# Diversity Evaluation of *Xylella fastidiosa* from Infected Olive Trees in Apulia (Southern Italy)

**DOI:** 10.5423/PPJ.OA.08.2015.0153

**Published:** 2016-04-01

**Authors:** Stefania M. Mang, Salvatore Frisullo, Hazem S. Elshafie, Ippolito Camele

**Affiliations:** 1 School of Agricultural, Forestry, Food and Environmental Sciences, University of Basilicata, Viale dell’Ateneo Lucano 10, 85100, Potenza, Italy; 2 Department of Agricultural, Food and Environmental Sciences, University of Foggia, Via Napoli 25, 71121, Foggia, Italy

**Keywords:** genetic variation, gyrase B, hypothetical protein, *Olea europea*, 16S rDNA gene

## Abstract

Olive culture is very important in the Mediterranean Basin. A severe outbreak of Olive Quick Decline Syndrome (OQDS) caused by *Xylella fastidiosa* infection was first noticed in 2013 on olive trees in the southern part of Apulia region (Lecce province, southern Italy). Studies were carried out for detection and diversity evaluation of the Apulian strain of *Xylella fastidiosa*. The presence of the pathogen in olive samples was detected by PCR amplifying the 16S rDNA, gyrase B subunit (*gyrB*) and HL hypothetical protein genes and single nucleotide polymorphisms (SNPs) assessment was performed to genotype *X. fastidiosa*. Twelve SNPs were recorded over *gyrB* and six SNPs were found for HL gene. Less variations were detected on 16S rDNA gene. Only *gyrB* and HL provided sufficient information for dividing the Apulian *X. fastidiosa* olive strains into subspecies. Using HL nucleotide sequences was possible to separate *X. fastidiosa* into subspecies *pauca* and *fastidiosa*. Whereas, nucleotide variation present on *gyrB* gene allowed separation of *X. fastidiosa* subsp. *pauca* from the other subspecies *multiplex* and *fastidiosa*. The *X. fastidiosa* strain from Apulia region was included into the subspecies *pauca* based on three genes phylogenetic analyses.

Olive (*Olea europea* L.) trees in 2007 have been cultivated worldwide over an area greater than 10 million hectares (IOC, 2009). The culture is primarily diffused in the Mediterranean Basin. Countries outside this area account for about 25% of the acreage but for only 10% of the entire production (Cimato and Attillio, 2011). Nowadays, Italian olive cultivation covers almost 2 million hectares, 80% of which are mostly located in southern Italy (Fontanazza, 2005). Recently, olive has been found infected by the *Xylella fastidiosa* Wells and Raju (1987) bacterium (Saponari et al., 2013) that caused the death of thousands of olive trees in Apulia (southern Italy). *Xylella fastidiosa* (*Xf* ) is a quarantine pathogen included in EPPO A 1 list, xylem-limited, Gram-negative bacterium that causes economically important plant diseases. More than 200 plant species are colonized by *Xf* including grapevine, coffee, citrus, almond, peach, plum, alfalfa, maple, olive, mulberry and also ornamentals such as oleander, sycamore, elm, oak and many other plants (Hopkins, 1989; Purcell and Hopkins, 1996; Simpson et al., 2000; Janse and Obradovic, 2010). It grows in the xylem of the hosts causing a wide variety of diseases such as Pierce’s Disease (PD) in grapevine, Citrus Variegated Chlorosis (CVC) in citrus, leaf scorch diseases in a broad range of plants including almond, coffee, sycamore, oleander, elm, pecan, pear, mulberry, maple and oak and other diseases of crops, ornamentals and woody plants (Hopkins and Purcell, 2002; Janse and Obradovic, 2010; Purcell, 2013). So far, four subspecies of the bacterium are known: *fastidiosa*, *multiplex*, *pauca* and *sandyi* (Schaad et al., 2004; Schuenzel et al., 2005; Randall et al., 2009). *Xf* subsp. *fastidiosa* comprises strains from grape, alfalfa and maple; *Xf* subsp. *multiplex* includes strains from plum, peach, elm, almond and sycamore; *Xf* subsp. *pauca* contains strains from citrus, coffee and recently those from olive and *Xf* subsp. *sandyi* which contains strains from oleander, daylily, magnolia and jacaranda (Hernandez-Martinez et al., 2007; Janse and Obradovic, 2010). Another subsp. of the same bacteria, from chitalpa, was proposed by Randall et al. (2009) and named *tashke*. This formed a distinct group from the other four subspecies previously mentioned but even today it still remains not fully accepted.

Until 2013, when a severe outbreak of *Xf* occurred in Apulia region (southern Italy) in the province of Lecce (Loconsole et al., 2014; Saponari et al., 2013), only a few reports of *Xf* infections on olive existed (Hernandez-Martinez et al., 2007; Wong et al., 2004) and the strain infecting olive was included in the subsp. *multiplex* or was classified as Genotype A by Chen et al. (2005). Initially, the etiology of the disease which occurred in the Apulia Region was unclear and the disorder was called “Olive Quick Decline Syndrome (OQDS)”. Afterwards, to understand the biology, genetics and phylogeny of *Xf* strain and subsequently to use all this information to better control this pathogen, a wide range of genes were investigated. Among these genes, one of the most studied and applied was the 16S rDNA gene, which can furnish precious data for *Xf* classification (Chen et al., 2000a; 2000b; Firrao and Bazzi, 1994; Martinati et al., 2007; Mehta and Rosato, 2001; Schaad et al., 2002) but evolves very slowly and thus, may have quite a minor resolution when used to infer relationships among closely related taxa. Therefore, other taxonomic markers were necessary for species identification such as the gene encoding the B subunit polypeptide of the DNA gyrase (*gyrB*). This was accepted to develop faster than the ribosomal RNA even preserving an elevated correlation with the whole genome homology (Murray et al., 2001; Rodriquez et al., 2003; Yamamoto and Harayama, 1995; 1996; Yamamoto et al., 1999). Both these previously described molecular markers along with the hypothetical protein HL gene (Francis et al., 2006) were utilized in this study to investigate the *Xf* strain from Apulia region. Sequence comparisons for 16S rDNA, *gyrB* and HL genes and phylogeny analyses were performed to explore the nucleotide diversity of the Apulian strain of *Xf* and to look over its relationships with other *Xf* taxa.

## Materials and Methods

### Biologic materials

In winter 2013–early spring 2014, in a Salento area, several samples of mature leaves and twigs were collected from olive trees showing scorch-like symptoms and/or yellowing. The samples were singly closed in double plastic bags, treated with insecticides, brought to the Department of Agricultural, Food and Environmental Sciences at the University of Foggia and subsequently processed in the laboratory of Plant Pathology, accredited to Apulia Region. After analysis, all samples were destroyed by double sterilization at 121°C for 20 min.

### DNA isolation

Genomic DNA (gDNA) was extracted from about 0.5–0.8 g of fresh tissue recovered from 5–10 mature leaf peduncles and midribs from symptomatic and healthy samples. Plant tissues were grinded in liquid nitrogen. Total nucleic acids isolation from homogenized plant tissue was performed with DNeasy plant mini kit (Qiagen, Heidelberg, Germany) according to the manufacturer’s instructions with some minor modifications. The extracted gDNA concentration was determined using an ND-1000 spectrophotometer (NanoDrop Technologies Inc., Wilmington, Delaware, U.S.A.) and then adjusted to 50–100 ng/μl. DNA samples were stored at −80°C for long term use.

### PCR and sequencing

Genomic DNA extracted from symptomatic and healthy olive samples was assessed by polymerase chain reaction (PCR) to detect the presence of the pathogen employing five pairs of species-specific primers: XF1/6, S-S-X.fas-0067-a-S-19/S-S-X.fas-0038-a-A-21, RST31/RST33, FXYgyr499/RXYgyr907 and HL5/6. The first three primers amplified 16S rDNA and RNA polymerase sigma-70 factor genes and the other gyrB gene and hypothetical protein gene (HL), respectively (Table 1). PCR conditions were identical to those reported in the original work of each author mentioned in Table 1. Each PCR reaction contained about 100 ng of gDNA template, 50 μl of 10× PCR Buffer (Invitrogen, Inc., New York, U.S.A.), 0.5 μM of each primer, 100 μM of each dNTP and 1U of *Taq* DNA polymerase (Invitrogen Inc., New York, U.S.A.) in a total reaction volume of 50 μl. Each assay was performed at least twice and a negative control (no gDNA template) was always included. Amplification products were visualized after electrophoresis in 1.2% agarose gel containing 0.1 μg/ml of ethidium bromide, run in 1X TBE buffer at 80V for 30 min and photographed. Subsequently, PCR amplicons were directly sequenced by BMR Genomics company (Padua, Italy), DNA sequences queried against NCBI database using the Basic Local Alignment Research Tool (BLAST) and megablast algorithm (Altschul et al., 1997), loaded and finally analyzed into MEGA v.6.0 program (Tamura et al., 2013).

### Sequences analysis, diversity assessment and phylogeny

Genetic diversity studies have been carried out on the *Xf* strains from infected olive trees in Apulia. In order to genotype the *Xf* from olive investigations on SNPs assessment on the three previous mentioned genes were done. All analyses were performed into MEGA6 phylogeny package where nucleotide sequences for each gene were aligned by ClustalW program (Tamura et al., 2013). Evolutionary divergence estimations were also performed in MEGA6 by Maximum Composite Likelihood (MCL) method (Tamura et al., 2004; 2013). Phylogeny reconstruction analyses were carried out using Neighbor-Joining (NJ) statistical method (Saitou and Nei, 1987). Branch support of the phylogenetic tree was tested by bootstrap method with 1000 replications (Felsenstein, 1985) and the nucleotide substitution model was adopted. The phylogenetic tree was inferred by a Kimura-2 Model algorithm (Kimura, 1980) with uniform rates among sites. Gaps/missing data were treated by complete deletion. For all investigations, gDNA nucleotide sequences of the *Xf* obtained in this study along with some others, downloaded from NCBI’s GenBank and used for comparative analyses, were employed (Table 2).

## Results

### DNA isolation, PCR and sequencing

About 50–100 ng/μl of gDNA was successfully isolated from infected olive trees and control healthy plants. Amplification of the above described genetic material was also successful and products of expected size were obtained for all genes investigated (Table 1) except for the negative controls. Thirty-four nucleotide sequences belonging to the 16S rDNA, gyrB and HL genes were obtained in this work and deposited into EMBL-EBI nucleotide archive (Table 2).

### Sequences analysis, diversity estimation and phylogeny 16S rDNA and RNA polymerase sigma-70 factor genes

**Fig. 1:**
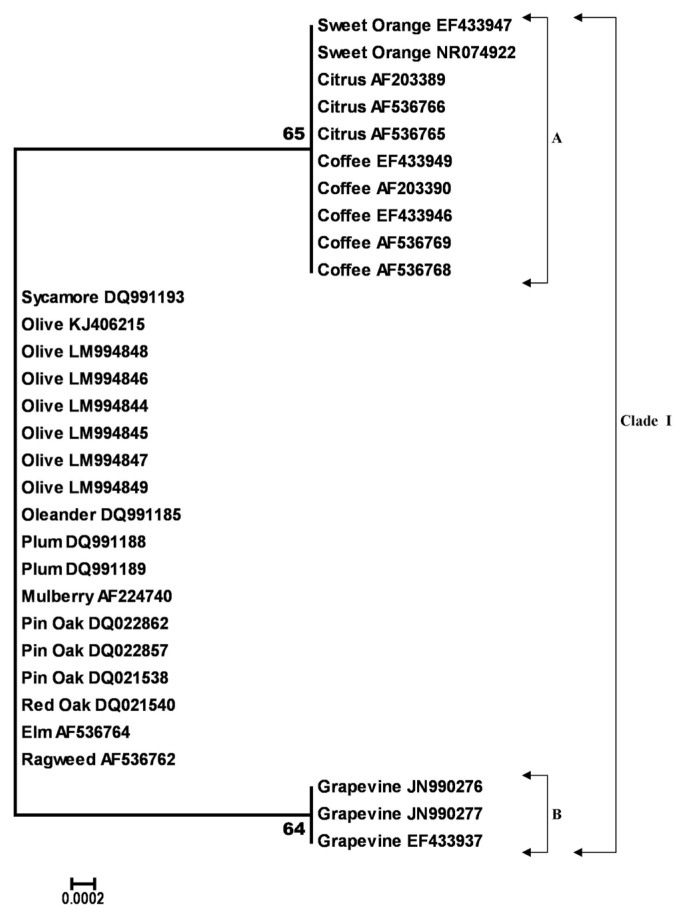
Neighbor-joining tree generated in MEGA6 from the 383 bp length alignment of 16S rDNA gene sequences of 31 *Xylella fastidiosa* specimens, using the Kimura-2 parameter model with uniform rates among sites, complete deletion gap handling and 1000-replication bootstrapping. Nodes with bootstrap values < 60% were eliminated. Bootstrap values are indicated next to relevant nodes. The tree is drawn to scale, with branch lengths in the same units as those of the evolutionary distances used to infer the phylogenetic tree.

The outcomes regarding diversity estimation and phylogeny were similar for the16S rDNA and RNA polymerase sigma-70 factor genes and therefore, only those referred to 16S rDNA gene are presented and discussed below. The final alignment of 16S rDNA gene contained 383 nucleotides. Out of these, 381 were conserved sites and only 2 variable. Two parsimony informative sites were found over the 16S rDNA sequence with no singletons. The average evolutionary divergence over all sequence pairs, computed using the MCL model was 0. All olive 16S rDNA sequences generated in this study shared a 100% identity with the olive strain OL-G2 of *Xf* (acc. no. KJ406215) and were also 100% identical to each other and to many *Xf* species and subspecies from various hosts (Fig. 1). Moreover, 16S rDNA gene showed a lower level of variation and only 2 SNPs were counted over a 383 bp length (data not shown). Analyzing the differences over 16S rDNA gene between *Xf* subsp. *pauca* strains from olive and those of the same subspecies from other hosts like coffee and citrus, we found only 1 diverse nucleotide. Quite a similar situation (1SNPs) was registered for nucleotide sequences of 16S rDNA of olive strains of *Xf* subsp. *pauca* and those from grape of *Xf* subsp. *fastidiosa*. The NJ phylogram of aligned and cured 16S rDNA sequences from 31 strains of *Xf* placed the majority of them into one single group (Clade I) divided into 2 subclades (A and B), with bootstrap values greater than 60% (Fig. 1). Subclade (A) grouped 16S rDNA gene sequences from olive along with those from oleander, plum, mulberry, elm, oak, pin oak, sycamore and ragweed which were placed together with no significant bootstrap support. Within this group, 16S rDNA gene sequences from coffee, citrus and sweet orange grouped together with a moderate bootstrap support of 65%. Subclade (B) grouped together the 16S rDNA gene sequences of grapevine strains with moderate bootstrap support (64%) as shown in Fig. 1. Overall, the high levels of nucleotide sequence similarity of *Xylella* strains from various hosts did not allowed us to separate them at subsp. level as shown by the single group (Fig. 1).

### Gyrase B gene

**Fig. 2:**
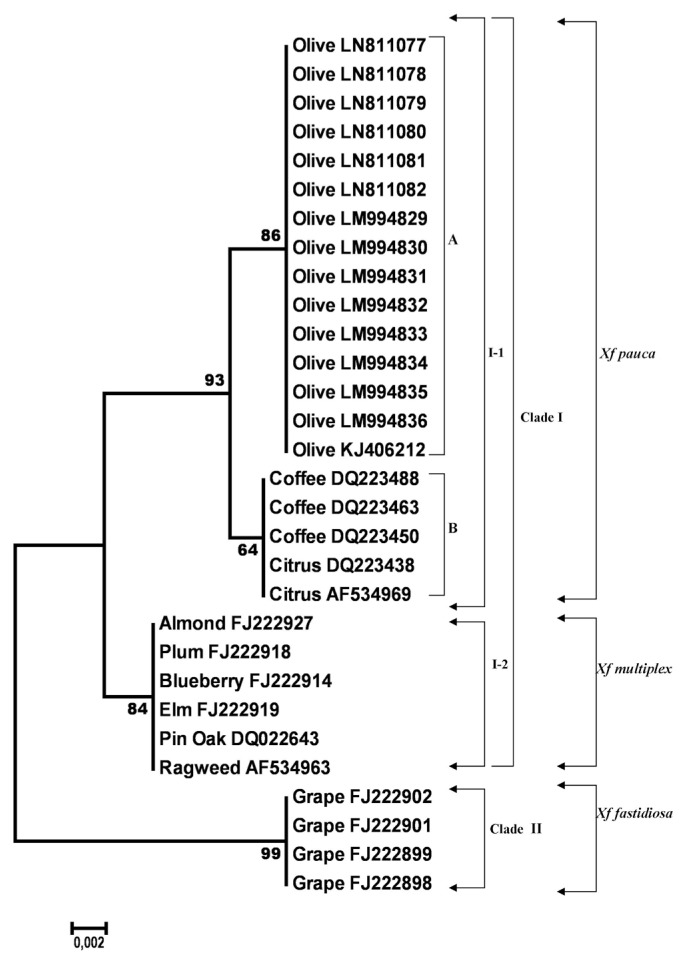
Neighbor-joining tree generated in MEGA6 from the 382 bp length alignment of gyrase B subunit gene sequences of 30 *Xylella fastidiosa* specimens, using the Kimura-2 parameter model with uniform rates among sites, complete deletion gap handling and 1000-replication bootstrapping. Nodes with bootstrap values inferior to 70% were eliminated. Bootstrap values are indicated next to relevant nodes. The tree is drawn to scale, with branch lengths in the same units as those of the evolutionary distances used to infer the phylogenetic tree.

The final alignment of *gyrB* gene sequences contained 30 sequences and included a total of 384 nucleotides. Out of these, 371 sites were conserved and 13 variable and parsimony-informative with no singletons. The average evolutionary divergence over all sequence pairs, computed using the MCL model (Tamura et al., 2004), was 0.01. All olive *gyrB* sequences generated in this study shared a 100% identity with the olive strain OL-G2 of *Xf* (acc. no. KJ406212) subsp. *pauca* originated from Italy and were also 100% identical to each other. Furthermore, the *gyrB* gene showed a high level of variation and 13 SNPs were counted over a 384 bp length (Table 3). Analyzing the differences between *Xf* strains from olive and those of the *Xf* subsp. *pauca* from coffee and citrus only 2 diverse nucleotides were found. Also, *gyrB* gene nucleotide sequences of *Xf* from olive, both from this study and one from GenBank, showed an insertion of two bases, which were never seen in all other *Xf* nucleotide sequences analyzed (data not shown). Among strains of *Xf* from olive and strains of *Xf* subsp. *multiplex* isolated from various hosts (almond, plum, blueberry, elm, ragweed and pin oak) 5 SNPs were registered. A different situation was registered for *Xf* nucleotide sequences of *gyrB* gene from olive and those of the same gene belonging to *Xf* subsp. *fastidiosa* from grape which were much more variable as 12 SNPs were detected. The NJ phylogram of aligned and cured *gyrB* gene sequences from 30 strains of *Xf* showed three clusters, supported with bootstrap values greater than 70% (Fig. 2). The first distinct cluster (I-1) was only formed by *Xf* isolates, known to belong to the subspecies *pauca* classified based on the already known nucleotide sequences from GenBank database, and supported by very high bootstrap values of 93%. Moreover, within this cluster two subgroups were detected, one (A) clustering together the *Xf* isolates from olive with a high bootstrap support (86%) and the other (B) grouping isolates of the same species from coffee and citrus supported by moderate bootstrap values of 64% (Fig. 2). The second distinct cluster (I-2), supported by very high bootstrap values (84%), grouped together strains of *X f* subsp. *multiplex* originated from various hosts. The third cluster (Clade II) supported by an excellent bootstrap of 99% and well separated from the other two previously described, grouped sequences of *X f* subsp. *fastidiosa* from grape (Fig. 2).

### Hypothetical protein gene

**Fig. 3:**
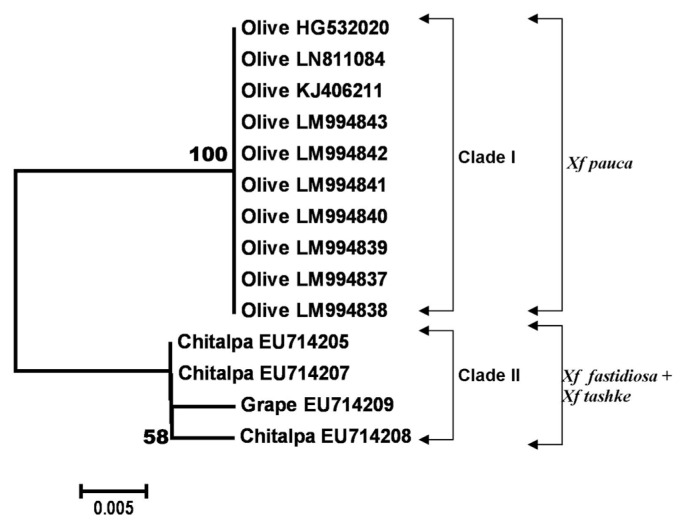
Neighbor-joining tree generated in MEGA6 from the 216 bp length alignment of hypothetical protein HL gene sequences of 14 *Xylella fastidiosa* specimens, using the Kimura 2-parameter model with uniform rates among sites, complete deletion gap handling and 1000-replication bootstrapping. Nodes with bootstrap values inferior to 30% were eliminated. Bootstrap values are indicated next to relevant nodes. The tree is drawn to scale, with branch lengths in the same units as those of the evolutionary distances used to infer the phylogenetic tree.

Despite a relatively high number of nucleotide sequences of *Xf* deposited for many genes, including those described before, a very low number of sequences of hypothetical protein (HL) still exist in all public nucleotide databases. All available sequences at the moment of the study were downloaded and analyzed along with the HL nucleotide sequences produced in this work. The final alignment of HL sequences included 14 sequences and contained a total of 216 nucleotides. A total number of 208 sites were conserved and 8 variable of which 6 were parsimony-informative and 2 singletons. The average evolutionary divergence over all sequence pairs using the MCL model (Tamura et al., 2004) was 0.01. Furthermore, olive HL sequences generated in this study were identical to each other and shared a 100% identity with two *Xf* strains present in GenBank (acc. numbers KJ406211 and HG532020, both originated from olive) and known to belong to the *Xf* subsp. *pauca*. The topology of the NJ tree based on HL gene showed that all HL nucleotide sequences from olive grouped together in a distinct cluster (Clade I). This clade (supported by an excellent bootstrap value of 100%) contained strains belonging to *Xf* subsp. *pauca* and the subspecies *pauca* was determined based on their 100% identity to the olive strains of *Xf* subsp. *pauca* already present in the NCBI GenBank (Fig. 3). Another distinct cluster (Clade II) was made by strains of *Xf* subsp. *tashke* from chitalpa (3 sequences) and one strain from grapevine which belongs to the subsp. *fastidiosa*, but less than 60% bootstrap support was registered within this cluster (Fig. 3). HL sequences from chitalpa and grape were almost identical except for one single base found in one sequence from chitalpa which can probably be a sequencing error. Results of HL nucleotide sequences obtained in this study confirm recent outcomes of Loconsole et al. (2014) who, detected *X. fastidiosa* in olive trees using molecular and serology methods and of Giampetruzzi et al. (2015) who, based on the draft genome sequence of *Xf* CoDiRO strain, classified *Xf* from olive into subsp. *pauca* group. Six SNPs were found over 216 bp length of HL nucleotide sequences (Table 4). Even if the length of the gene investigated is quite small, a high diversity was recorded and the SNPs found resulted associated with two types corresponding to different hosts (Table 4). Specific SNPs were only found for olive (Type I) but not for the other two host plants, chitalpa and grape (Type II) as shown in Table 4. The SNPs discovered showed that no association exists between them and phylogenetic subgroups corresponding to an *Xf* subspecies since strains from two different subspecies e.g. *Xf* subsp. *tashke* and *Xf* subsp. *fastidiosa* were grouped together (Type II) (Table 4).

## Discussion

Diversity and phylogenetic studies carried out on *Xf* from Apulian olive trees gave us comparable results with the recent studies (Carridi et al., 2014; Elbeaino et al., 2014; Loconsole et al., 2014; Saponari et al., 2013). Phylogenetic trees had slightly different topologies for the three genes investigated although some clusters were maintained in trees originated from data of *gyrB* and HL genes. For the 16S rDNA gene the distinction between the *Xf* subsp. was unclear since a unique group containing all *Xf* strains was observed (Fig. 1). Our results of this gene are concordant with what was previously reported by Mehta and Rosato (2001) who demonstrated that the 16S rDNA sequences of *Xf* from various host plants, were very similar and a small number of nucleotide substitutions (2 to 4) were only detected in grapevine and plum, also showing that a low level of divergence over 16S rDNA sequences exists. Data from this study also support the results of Chen et al. (2000b) which observed higher levels of heterogeneity in 16S rDNA gene only when *Xf* was compared to same gene from *Xanthomonas* and *Stenotrophomonas*.

Other genes explored in this study like *gyrB* and HL furnished enough nucleotide variation to identify and classify our *Xf* strains from olive (Figs. 2 and 3). Findings from this study obtained with *gyrB* gene are concordant with those of Cariddi et al. (2014) which proved the separation of three subsp. of *Xf* based on the variation which exists on this gene, even if in their study a single *gyrB* gene sequence from olive (strain OL-G2) and a smaller number of different gyrB gene sequences from other hosts were considered. Furthermore, SNPs identified, in our work, over the *gyrB* gene sequence grouped together strains of *Xf* subsp. *pauca* into 2 types: type I containing strains from olive and type II harvesting strains of the same subspecies from coffee and citrus. Strains belonging to the *Xf* subsp. *multiplex* grouped together (type III) based on their SNPs revealed over *gyrB* gene independently of their host plant origin. Another type named IV containing strains of *Xf* subsp. *fastidiosa* from grape was also recorded (Table 3). Only two types, based on the SNPs counted on HL gene, harvesting strains of *Xf* from olives (type I) and chitalpa (type II) were found, but this can be explained by the small number of sequences analyzed for this gene and if we could increase their number probably more types could be observed as it was seen for the *gyrB* gene (Tables 3 and 4).

SNPs analyses for both gyrase B and HL genes showed that olive sequences obtained in this study were identical among them and very similar to the olive *Xf* sequences already present into GenBank.

Both gyrB and HL genes, were initially employed to detect the presence of *Xf* (Francis et al., 2006; Rodrigues et al., 2003) and recently used for a preliminary molecular identification of *Xf* in olives (Saponari et al., 2013). Only one of these (*gyrB*) was employed to detect the presence of a *Xf* strain infecting olive and oleander (Carridi et al., 2014) but can be also useful to classify the *Xf* pathogen at subsp. level.

The epidemic nature of OQDS disease induced by *Xf* susp. *pauca* strain CoDiRO (Carridi et al., 2014) on olives in Apulia region and in particular in Lecce province, was already reported and considered to be a consequence of the globalization in the plant movement (Catalano, 2015). Very recently, Nunney et al. (2014) reported a strain of *Xf* subsp. *pauca* in Costa Rica on oleander, but in Central America this particular strain was never found on citrus or grape and Costa Rica could be its centre of origin. The same authors also suggest the possible existence of some unknown genetic forms of *Xf* in South America. They warn us that the formation of novel genetic forms through inter-subspecific recombination could be of great importance because they could be introduced into other regions through plant movement, and as a consequence they may attack commercially important crops (Elbeaino et al., 2014; Nunney et al., 2014). *Xf* subsp. *pauca* found in the Salento area is highly similar to *Xf* subsp. *pauca* found in Costa Rica. This strain is diverse from other isolates belonging to subspecies *pauca*, in fact, for example, the CoDiRO strain does not infect citrus as other *pauca* isolates do. It is very possible that in the near future the CoDiRO strain will be classified as a distinct and new subspecies of *Xf*.

## Figures and Tables

**Table 1:** PCR primers used in this study to detect *X. fastidiosa* in infected olive trees and their relative target genes, PCR product size and references

Target Gene	Primers pairs	Oligonucloetide sequence (5′→3′)	Approx. amplicon size (bp)	References
16S rDNA	XF1-F	CAGCACATTGGTAGTAATAC	404	Firrao and Bazzi, 1994
	XF6-R	ACTAGGTATTAACCAATTGC		

16S rDNA	S-S-X.fas-0067-a-S-19	CGG CAG CAC ATT GGT AGT A	603	Rodrigues et al., 2003
	S-S-X.fas-0038-a-A-21	CGA TAC TGA GTG CCA ATT TGC		

RNA polymerase sigma factor	RST31	GCGTTAATTTTCGAAGTGATTCGATTGC	733	Minsavage et al., 1994
	RST33	CACCATTCGTATCCCGGTG		

Gyrase B	FXYgyr499	CAG TTA GGG GTG TCA GCG	420	Rodrigues et al., 2003
	RXY gyr907	CTC AAT GTA ATT ACC CAA GGT		

Hypothetical protein (HL)	HL5	AAGGCAATAAACGCGCACTA	221	Francis et al., 2006
	HL6	GGTTTTGCTGACTGGCAACA		

**Table 2:** List of *X. fastidiosa* taxa used in this study along with their host common names, geographical origin, locus and their GenBank accession numbers with sources

Isolate/strain/clone of *X. fastidiosa*	Host common name	Geographical origin	Locus		GenBank accession no./Source		
*X. fastidiosa* isolate 1408	Olive	Italy	16S	rDNA	LM994844	(this	study)
*X. fastidiosa* isolate 1423	Olive	Italy	〃	〃	LM994845	〃	〃
*X. fastidiosa* isolate 1433	Olive	Italy	〃	〃	LM994846	〃	〃
*X. fastidiosa* isolate 1440	Olive	Italy	〃	〃	LM994847	〃	〃
*X. fastidiosa* isolate 1443	Olive	Italy	〃	〃	LM994848	〃	〃
*X. fastidiosa* isolate 1444	Olive	Italy	〃	〃	LM994849	〃	〃
*X. fastidiosa* isolate OL-G2	Olive	Italy	〃	〃	KJ406215	(NCBI	GenBank)
*X. fastidiosa* strain SLS55	Sycamore	–	〃	〃	DQ991193	〃	〃
*X. fastidiosa* strain GH-9	Oleander	–	〃	〃	DQ991185	〃	〃
*X. fastidiosa* isolate P3	Coffee	Brazil	〃	〃	AF536769	〃	〃
*X. fastidiosa* isolate CRS2	Coffee	Brazil	〃	〃	AF536768	〃	〃
*X. fastidiosa* strain CO.01	Coffee	–	〃	〃	AF203390	〃	〃
*X. fastidiosa* isolate Cafe 20-11	Coffee	Brazil	〃	〃	EF433946	〃	〃
*X. fastidiosa* isolate CaVIc2	Coffee	Costa Rica	〃	〃	EF433949	〃	〃
*X. fastidiosa* isolate SL1	Citrus	Brazil	〃	〃	AF536766	〃	〃
*X. fastidiosa* strain CI.52	Citrus	–	〃	〃	AF203389	〃	〃
*X. fastidiosa* isolate B14	Citrus	Brazil	〃	〃	AF536765	〃	〃
*X. fastidiosa* isolate Taq30	Sweet orange	Brazil	〃	〃	EF433947	〃	〃
*X. fastidiosa* clone 9a6c	Sweet orange	Brazil	〃	〃	NR074922	〃	〃
*X. fastidiosa* strain 2–5	Plum	–	〃	〃	DQ991188	〃	〃
*X. fastidiosa* strain 2–4	Plum	–	〃	〃	DQ991189	〃	〃
*X.fastidiosa* isolate MUL-1	Mulberry	USA	〃	〃	AF224740	〃	〃
*X. fastidiosa* isolate ELM-1	Elm	USA	〃	〃	AF536764	〃	〃
*X. fastidiosa* clone PODon	Pine Oak	USA	〃	〃	DQ022862	〃	〃
*X. fastidiosa* clone RO1	Red Oak	USA	〃	〃	DQ021540	〃	〃
*X.fastidiosa* clone 138H	Pine Oak	USA	〃	〃	DQ022857	〃	〃
*X. fastidiosa* clone POLime	Pine Oak	USA	〃	〃	DQ021538	〃	〃
*X. fastidiosa* isolateRGW-R	Ragweed	USA	〃	〃	AF536762	〃	〃
*X. fastidiosa* isolate VvIIc1	Grapevine	Costa Rica	〃	〃	EF433937	〃	〃
*X. fastidiosa* strain GV102	Grapevine	Taiwan	〃	〃	JN990276	〃	〃
*X. fastidiosa* strain GV103	Grapevine	Taiwan	〃	〃	JN990277	〃	〃
*X. fastidiosa* isolate 1408	Olive	Italy	RNA pol sigma factor		HG941632	(this	study)
*X. fastidiosa* isolate 1409	Olive	Italy	〃	〃	HG941633	〃	〃
*X. fastidiosa* isolate 1422	Olive	Italy	〃	〃	HG941634	〃	〃
*X. fastidiosa* isolate 1423	Olive	Italy	〃	〃	HG941635	〃	〃
*X. fastidiosa* isolate 1426	Olive	Italy	〃	〃	HG941636	〃	〃
*X. fastidiosa* isolate 1433	Olive	Italy	〃	〃	HG941637	〃	〃
*X. fastidiosa* strain N. 1408	Olive	Italy	Gyrase-B		LM994829	〃	〃
*Xylella fastidiosa* strain N. 1408	Olive	Italy	〃	〃	LM994830	〃	〃
*X. fastidiosa* strain N. 1423	Olive	Italy	〃	〃	LM994831	〃	〃
*X. fastidiosa* strain N. 1433	Olive	Italy	〃	〃	LM994832	〃	〃
*X. fastidiosa* strain N. 1440	Olive	Italy	〃	〃	LM994833	〃	〃
*X. fastidiosa* strain N. 1444	Olive	Italy	〃	〃	LM994834	〃	〃
*X. fastidiosa* strain N. 1445	Olive	Italy	〃	〃	LM994835	〃	〃
*X. fastidiosa* strain N. 1463	Olive	Italy	〃	〃	LM994836	〃	〃
*X. fastidiosa* strain N. 1499	Olive	Italy	〃	〃	LN811077	〃	〃
*X. fastidiosa* strain N. 1500	Olive	Italy	〃	〃	LN811078	〃	〃
*X. fastidiosa* strain N. 1501	Olive	Italy	〃	〃	LN811079	〃	〃
*X. fastidiosa* strain N. 1505	Olive	Italy	〃	〃	LN811080	〃	〃
*X. fastidiosa* strain N. 1506	Olive	Italy	〃	〃	LN811081	〃	〃
*X. fastidiosa* strain N. 1508	Olive	Italy	〃	〃	LN811082	〃	〃
*X. fastidiosa* strain Olive	Olive	Italy	〃	〃	LN811083	(NCBI	GenBank)
*X. fastidiosa* isolate OL-G2	Olive	Italy	〃	〃	KJ406212	〃	〃
*X. fastidiosa* isolate SL 1	Citrus	Brazil	〃	〃	AF534969	〃	〃
*X. fastidiosa* strain CIT-J8	Citrus	Brazil	〃	〃	DQ223438	〃	〃
*X. fastidiosa* strain COF-E21	Coffee	Brazil	〃	〃	DQ223450	〃	〃
*X. fastidiosa* strain COF-E10	Coffee	Brazil	〃	〃	DQ223463	〃	〃
*X. fastidiosa* strain COF-J62	Coffee	Brazil	〃	〃	DQ223488	〃	〃
*X. fastidiosa* isolate P3	Coffee	Brazil	〃	〃	AF534972	〃	〃
*X. fastidiosa* strain Georgia 1	Grape	USA	〃	〃	FJ222898	〃	〃
*X. fastidiosa* strain Mus 1	Grape	USA	〃	〃	FJ222901	〃	〃
*X. fastidiosa* strain Kingsburg	Grape	USA	〃	〃	FJ222902	〃	〃
*X. fastidiosa* strain M12	Almond	USA	〃	〃	FJ222927	〃	〃
*X. fastidiosa* strain GA Plm19b	Plum	USA	〃	〃	F J222918	〃	〃
*X. fastidiosa* strain Elm	Elm	USA	〃	〃	FJ222919	〃	〃
*X. fastidiosa* strain BB4	Blueberry	USA	〃	〃	FJ222914	〃	〃
*X. fastidiosa* strain RGW-R	Ragweed	USA	〃	〃	AF534963	〃	〃
*X. fastidiosa* clone 109H	Pin Oak	USA	〃	〃	DQ022643	〃	〃
*X. fastidiosa* isolate 1422	Olive	Italy	HL (hypothetical protein)		LM994837	(this	study)
*X. fastidiosa* isolate 1423	Olive	Italy	〃	〃	LM994838	〃	〃
*X. fastidiosa* isolate 1433	Olive	Italy	〃	〃	LM994839	〃	〃
*X. fastidiosa* isolate 1440	Olive	Italy	〃	〃	LM994840	〃	〃
*X. fastidiosa* isolate 1443	Olive	Italy	〃	〃	LM994841	〃	〃
*X. fastidiosa* isolate 1444	Olive	Italy	〃	〃	LM994842	〃	〃
*X. fastidiosa* isolate 1445	Olive	Italy	〃	〃	LM994843	〃	〃
*X. fastidiosa* isolate 1507	Olive	Italy	〃	〃	LN811084	〃	〃
*X. fastidiosa* isolate OL-1	Olive	Italy	〃	〃	HG532020	(NCBI	GenBank)
*X. fastidiosa* isolate OL-G2	Olive	Italy	〃	〃	KJ406211	〃	〃
*X. fastidiosa* isolate NM01	Chitalpa	USA	〃	〃	EU714205	〃	〃
*X. fastidiosa* isolate AZ03	Chitalpa	USA	〃	〃	EU714207	〃	〃
*X.fastidiosa* isolate AZ04	Chitalpa	USA	〃	〃	EU714208	〃	〃
*X. fastidiosa* isolate NM Grape 01	Grapevine	USA	〃	〃	EU714209	〃	〃

Note: no data are reported by “–”

**Table 3:** Single nucleotide polymorphism (SNP) sites registered over 384 base pairs length of *X. fastidiosa* gyrase subunit B gene DNA sequences found among 30 specimens

Types	Species and subspecies	Host common name	Nucleotide position (No.)												

			25	89	127	175	193	200	217	220	223	249	322	364	376
I	*X. fastidiosa* subsp. *pauca*	Olive	**A**	**C**	**T**	**G**	**G**	**T**	**T**	**C**	**T**	**T**	**T**	**C**	**A**
II	*X. fastidiosa* subsp. *pauca*	Coffee	**A**	**A**	**T**	**G**	**G**	**T**	**T**	**T**	**T**	**T**	**T**	**C**	**C**
II	*X. fastidiosa* subsp. *pauca*	Citrus	**A**	**A**	**T**	**G**	**G**	**T**	**T**	**T**	**T**	**T**	**T**	**C**	**C**
III	*X. fastidiosa* subsp. *multiplex*	Almond	**G**	**A**	**C**	**G**	**G**	**T**	**C**	**C**	**T**	**T**	**T**	**C**	**C**
III	*X. fastidiosa* subsp. *multiplex*	Plum	**G**	**A**	**C**	**G**	**G**	**T**	**C**	**C**	**T**	**T**	**T**	**C**	**C**
III	*X. fastidiosa* subsp. *multiplex*	Blueberry	**G**	**A**	**C**	**G**	**G**	**T**	**C**	**C**	**T**	**T**	**T**	**C**	**C**
III	*X. fastidiosa* subsp. *multiplex*	Ragweed	**G**	**A**	**C**	**G**	**G**	**T**	**C**	**C**	**T**	**T**	**T**	**C**	**C**
III	*X. fastidiosa* subsp. *multiplex*	Elm	**G**	**A**	**C**	**G**	**G**	**T**	**C**	**C**	**T**	**T**	**T**	**C**	**C**
III	*X. fastidiosa* subsp. *multiplex*	Pin Oak	**G**	**A**	**C**	**G**	**G**	**T**	**C**	**C**	**T**	**T**	**T**	**C**	**C**
IV	*X. fastidiosa* subsp. *fastidiosa*	Grape	**G**	**A**	**T**	**A**	**A**	**C**	**C**	**A**	**G**	**C**	**C**	**G**	**C**

**Table 4:** Single nucleotide polymorphisms (SNPs) registered over 216 base pairs length of *X. fastidiosa* hypothetical protein gene DNA sequences found among 14 specimens

Types	Species and subspecies	Host common name	Nucleotide position (No.)					

			31	69	72	82	174	180
I	*X. fastidiosa* subsp. *pauca*	Olive	**C**	**T**	**A**	**T**	**G**	**C**
II	*X. fastidiosa* subsp. *tashke*	Chitalpa	**T**	**C**	**G**	**C**	**A**	**G**
II	*X. fastidiosa* subsp. *fastidiosa*	Grape	**T**	**C**	**G**	**C**	**A**	**G**
